# Iterative Methods for Obtaining Energy-Minimizing Parametric Snakes with Applications to Medical Imaging

**DOI:** 10.1155/2012/918510

**Published:** 2012-03-04

**Authors:** Alexandru Ioan Mitrea, Radu Badea, Delia Mitrea, Sergiu Nedevschi, Paulina Mitrea, Dumitru Mircea Ivan, Octavian Mircia Gurzău

**Affiliations:** ^1^Department of Mathematics, Technical University of Cluj-Napoca, George Baritiu Street, no. 25, 400020 Cluj-Napoca, Romania; ^2^Department of Ultrasonography, University of Medicine and Pharmacy “Iuliu Haţieganu” Cluj-Napoca, Victor Babeş Street, no. 8, 400079 Cluj-Napoca, Romania; ^3^Department of Computer Science, Technical University of Cluj-Napoca, George Baritiu Street, no. 26-28, 400027 Cluj-Napoca, Romania

## Abstract

After a brief survey on the parametric deformable models, we develop an iterative method based on the finite difference schemes in order to obtain energy-minimizing snakes. We estimate the approximation error, the residue, and the truncature error related to the corresponding algorithm, then we discuss its convergence, consistency, and stability. Some aspects regarding the prosthetic sugical methods that implement the above numerical methods are also pointed out.

## 1. Introduction

The deformable models represent a powerful researched model-based approach to computer-assisted medical image analysis, their applications in this framework including image segmentation, shape representation and motion tracking. The theory of deformable models is an interdisciplinary scientific domain, which has appeared and developed in the last two decades, in strong connection with practical problems of medicine, image processing, and physics. This theory joins methods, results, and techniques of various mathematical fields, physics and mechanics. The mathematical foundation of this theory represents the confluence of Functional Analysis, Approximation Theory, Differential Equations, Differential Geometry, Calculus of Variations, Numerical Analysis, Linear Algebra, and Probability Theory. The ancestors of the deformable models, in classical sense, are considered Fischler and Elschlager, with their spring-loaded templates, [1], together with Widrow [2], with its rubber mask technique [2, 3].

The theory of deformable models, in its modern form, originates from the general theory of continuous multidimensional deformable models in a Lagrangian dynamic of Terzopoulos (1987) [4]. In fact, the deformable curves (2D models) and the deformable surfaces (3D models) gained popularity after their use in computer vision by Kaas et al. [5] and in computer graphics, by Terzopoulos and Fleischer [6] in the mid-1980s. Since then, the deformable models, known also as active contour models or snakes, have been extensively used for many applications both in 2D and 3D.

Two general types of deformable models have been developed: firstly, the parametric or variational models, which originate from the papers of Kaas et al. [5] and are based on the minimization of the energy-functional associated to the model, and secondly, the geometric models, which were introduced independently by Caselles et al. [7] and Malladi et al. [8], and are based on the front propagation theory [9].

A good survey on deformable models and their applications can be found in [10, 11]. Recent contributions on parametric deformable models have appeared in the papers [12, 13]. On the general topic of numerical methods applied in medical imaging, the recent papers [14, 15] must be mentioned.

In this paper, we deal with the deformable parametric models. The basic goal of the theory of parametric deformable models is to determine the energy-minimizing 2D or 3D models, namely, the curves or surfaces which minimize the corresponding energy functional. Two approaches will point out in order to obtain the optimal model. The first approach is based on the Euler-Lagrange-Poisson (ELP) and Euler-Gauss-Ostrogradski (EGO) equations of Calculus of Variations in order to minimize the energy-functional. The second one (the classical approach) consists of using reconstruction methods, such as the interpolation of the sparse data extracted from the image, in order to obtain a representation of the original data. In what follows we develop methods and techniques related to the first approach. Generally, the energy-functional is not convex, so it may have many local minimum. On the other hand, the analytic solution of (ELP) equation has a complicated form or it is inaccessible explicitly. Therefore, a practical and strong approach for finding local minimum of the energy functional is to construct a dynamic system that is governed by the energy functional and allow the system to evolve to the equilibrium state. Dynamic models are valuable for medical image analysis, because most anatomical structures are deformable and continually undergo nonrigid motion “in vivo.” In fact, the user is interested to find a good 2D or 3D contour in a given area. Consequently, a rough prior estimation of the 2D or 3D model is provided, then this initial model undergoes a deformation until reaching a local minimum of the energy functional. This deformation process can be achieved in one of the following ways:

1. in a Hamiltonian-type approach, by performing a strictly decreasing energy path, for example, via dynamic programming methods [16, 17];
2. in a Lagrange-type approach, by applying the mechanical principles of Lagrange [3, 18];
3. by using a friction force, in order to constrain the displacement of the snake [5];
4. by using the (ELP) evolution equation, associated to the initial (ELP) equation [19].

In this paper, we shall adopt the method of the evolution equation. So, a prior estimation of the deformable surface is provided, then it is refined step by step, based on the (EGO) equation and using discretization methods.

The paper outline is as follows. The next section is devoted to present 2D and 3D energy-minimizing models, both in their static and dynamic forms. The method for reducing the 3D problem to a 2D modeling is also pointed out, in order to minimize the computational costs of the numerical methods. The third section contains the main theoretical result of the paper. Based on finite difference schemes of explicit type, we derive an (ELP) algorithm for obtaining an energy-minimizing snake in its approximated form, then we estimate its approximation error and we discuss its consistency, convergence, and stability. The last section deals with the behaviour of prosthetic surgical methods and prosthetic medical materials, based on Software tools, which implement the iterative methods developed in the previous sections.

## 2. Energy-Minimizing Models

### 2.1. Energy-Minimizing Snakes (2D Models)

From mathematical point of view, a 2D parametric deformable model (usually known as *snake*) is provided by a family *𝒜* of parametrized smooth curves satisfying given boundary conditions and an associated energy-functional. More exactly, denote by *C* ^2^([0,1], ℝ^2^) the space of all vectorial functions *v* = (*x*,*y*)^*T*^ so that the scalar functions *x* = *x*(*s*) and *y* = *y*(*s*), 0 ≤ *s* ≤ 1 are continuous together with their derivatives up to the second-order on the standard interval [0,1], that is, *x*, *y* ∈ *C* ^2^[0,1]; obviously, we can consider an arbitrary compact interval [*a*, *b*] of the real axis instead of [0,1]. The family *𝒜* of *admissible deformations* consists of all parametrized curves (snakes):

(1) $$\begin{array}{cc} \left(\gamma \right):v\left(s\right)={\left(x\left(s\right),y\left(s\right)\right)}^{T}, & \\ 0\;\leq s\leq 1,\;v\in C^{2}\left(\left[0,1\right],{\mathbb{R}}^{2}\right), & \end{array}$$

such that the values *v*(0), *v*(1), *v*′(0), and *v*′(1) are given; we adopt the notation |*v*|^2^ = |*x*|^2^ + |*y*|^2^.

In order to find the optimal position of the snake, it is necessary to characterize its state, by means of an *energy-functional, *that is associated to the class *𝒜*. Let us consider the following data:

- the *weight-functions w*_1_(*s*) and *w*_2_(*s*), which control the elasticity and the rigidity of (*γ*), respectively; generally, these are nonnegative scalar functions of class *C* ^2^[0,1],
- the *image intensity* function *I* = *I*(*x*, *y*), which is a real function of class *C* ^2^(ℝ^2^),
- the *potential* associated to the external forces, represented by a real function *P*(*v*) = *P*(*x*, *y*), of class *C* ^2^(ℝ^2^). The simplest useful choice for the potential is *P*(*v*) = *w*_3_ *I*(*v*), where *w*_3_ is a weight-scalar. The most used choice is *P*(*v*) = −*λ*|∇*I*(*v*)|, where *λ* > 0 is a given scalar and ∇ = (∂/∂*x*,∂/∂*y*)^*T*^ is the Hamilton (nabla) operator; this choice will be used in this paper, too. Note that *P* can be defined also by *P* = *G*_*σ*_0__∗*I*, that is, the Gaussian (variance *σ*_0_) filtered image of the input image *I* [10], and
- the vectorial function *k*(*v*) = (*k*_1_(*v*),*k*_2_(*v*))^*T*^ of class *C* ^1^(ℝ^2^, ℝ^2^) which control the local dilatation or local contraction of (*γ*) along its normal; usually, we take *k*(*v*) = *cv*, with *c* ∈ ℝ.

The shape of the snake (*γ*) subject to the image *I*(*v*) is dictated by the *energy functional:*

(2) $$E\left(v\right)=E_{\mathrm{int}}\left(v\right)+E_{\text{ext}}\left(v\right)+E_{\text{bal}}\left(v\right),$$

where the terms of the right hand of (2) are defined as follows.

The *internal energy*

(3) $$E_{\mathrm{int}}\left(v\right)=E_{\text{els}}\left(v\right)+E_{\text{rig}}\left(v\right)$$

is obtained by adding the *elastic energy*

(4) $$E_{\text{els}}\left(v\right)=\overset{1}{\underset{0}{\int }}\alpha \left(s\right){\left|v^{\prime }\left(s\right)\right|}^{2}\text{d}s,$$

and the *rigid (bending) energy*

(5) $$E_{\text{rig}}\left(v\right)=\overset{1}{\underset{0}{\int }}\beta \left(s\right){\left|v^{\prime \prime }\left(s\right)\right|}^{2}\text{ds}.$$

The internal energy characterizes the deformation of a stretchy, flexible snake (contour). The values of *w*_1_(*s*) and *w*_2_(*s*) show the extent to which the snake can stretch or bend at an arbitrary point (*x*(*s*), *y*(*s*)) of the snake.

The *external energy, *derived from the image, is given by

(6) $$E_{\text{ext}}\left(v\right)=\overset{1}{\underset{0}{\int }}P\left(v\left(s\right)\right)\text{d}s=-\lambda \overset{1}{\underset{0}{\int }}{\left|\nabla I\left(x,y\right)\right|}^{2}\text{ds},$$

and it allows to find the edges in an image so that the snake is attracted to contour with large image gradients.

The *balloon energy *is an energy of constrained-type, defined as

(7) $$E_{\text{bal}}\left(v\right)=-\overset{1}{\underset{0}{\int }}\det\left(k\left(v\right),v^{\prime }\right)\text{ds}=-\overset{1}{\underset{0}{\int }}\left(k_{1}y^{\prime }-k_{2}x^{\prime }\right)\text{ds}.$$

This energy can be added, optionally, by users, in order to expand (or contract) the snake.

Denoting by

(8) $$\begin{array}{cc} F\left(s,v,v^{\prime },v^{\prime \prime }\right) & =w_{1}\left(s\right)\left|v^{\prime 2}\left(s\right)\right|+w_{2}\left(s\right)\left|v^{\prime \prime 2}\left(s\right)\right|\\ & \;+P\left(v\left(s\right)\right)-\det\left(k\left(v\right),v^{\prime }\right),\;\\ P\left(v\right) & =-\lambda {\left|\nabla I\left(v\right)\right|}^{2}, \end{array}$$

the following expression of energy-functional *E*(*v*) is obtained from (2)–(8):

(9) $$E\left(v\right)=\overset{1}{\underset{0}{\int }}F\left(s,v,v^{\prime },v^{\prime \prime }\right)\text{ds}.$$

By definition, the triple (*𝒜*, *I*, *E*) is said to be a *deformable 2D model (snake).*

The basic goal of a deformable parametric model is to minimize its energy-functional *E*(*v*), which leads to the energy-minimizing snake. The minimization of the snake energy gives rise to the following vectorial Euler-Lagrange-Poisson (ELP) Equation of Calculus of Variations:

(10) $$\frac{\partial F}{\partial v}-\frac{d}{ds}\left(\frac{\partial F}{\partial v^{\prime }}\right)+\frac{d^{2}}{ds^{2}}\left(\frac{\partial F}{\partial v^{\prime \prime }}\right)=0.$$

Now, taking into account the relations (8) and (10), we obtain the vectorial (ELP) equation:

(11) $$\begin{array}{cc} & 2w_{2}\left(s\right)v^{\text{iv}}\left(s\right)+4w_{2}^{\prime }\left(s\right)v^{\prime \prime \prime }\left(s\right)+2\left[w_{2}^{\prime \prime }\left(s\right)-w_{1}\left(s\right)\right]v^{\prime \prime }\left(s\right)\\ & \;\;-\left[2w_{1}^{\prime }\left(s\right)I_{2}+\mathrm{Tr}\left(\nabla k\right)J_{2}\right]v^{\prime }\left(s\right)+\nabla P\left(v\left(s\right)\right)=0, \end{array}$$

where $I_{2}=\left(\begin{array}{cc} 1 & 0\\ 0 & 1 \end{array}\right)$, $J_{2}=\left(\begin{array}{cc} 0 & 1\\ -1 & 0 \end{array}\right)$, and *Tr*⁡(*A*) is the trace of a square matrix *A*.

The scalar (ELP) equations, derived from (11), have the form:

(12) $$\begin{array}{cc} & 2w_{2}x^{\text{iv}}+4w_{2}^{\prime }x^{\prime \prime \prime }+2\left(w_{2}^{\prime \prime }-w_{1}\right)x^{\prime \prime }-2w_{1}^{\prime }x^{\prime }\\ & \;\;-\left(\frac{\partial k_{1}}{\partial x}+\frac{\partial k_{2}}{\partial y}\right)y^{\prime }+\frac{\partial P}{\partial x}=0,\\ & 2w_{2}y^{\text{iv}}+4w_{2}^{\prime }y^{\prime \prime \prime }+2\left(w_{2}^{\prime \prime }-w_{1}\right)y^{\prime \prime }-2w_{1}^{\prime }y^{\prime }\\ & \;\;+\left(\frac{\partial k_{1}}{\partial x}+\frac{\partial k_{2}}{\partial y}\right)x^{\prime }+\frac{\partial P}{\partial y}=0. \end{array}$$

On the other hand, we infer from(8)

(13) $$\begin{array}{cc} F\left(s,v,v^{\prime },v^{\prime \prime }\right) & =w_{1}\left(x^{\prime 2}+y^{\prime 2}\right)+w_{2}\left(x^{\prime \prime 2}+y^{\prime \prime 2}\right)\\ & \;+P\left(x,y\right)-k_{1}y^{\prime }+k_{2}x^{\prime }, \end{array}$$

which leads to

(14) $$\frac{\partial^{2}F}{\partial {\left(v^{\prime \prime }\right)}^{2}}={\left(2w_{1},2w_{2}\right)}^{T}.$$

According to the Legendre conditions and the hypothesis *w*_2_ > 0, the relation (14) proves that any solution of the (ELP) equations (11) or (12) provides a minimum for the energy-functional *E*(*v*), namely, an energy-minimizing snake.

**Example 1**

If we choose in (12) *w*_1_ = 1, *w*_2_ = 0.05, and the boundary conditions *v*(0) = *v*(1) = (0,5)^*T*^, *v*′(0) = *v*′(1) = (0.5,0.5)^*T*^ we obtain the general solution of the (ELP) equation:

(15) $$\begin{array}{c} x\left(s\right)=C_{1}e^{3.8042s}+C_{2}e^{-3.8042s}+C_{3}e^{2.3511s}+C_{4}e^{-2.3511s},\\ y\left(s\right)=C_{5}e^{3.8042s}+C_{6}e^{-3.8042s}+C_{7}e^{2.3511s}+C_{8}e^{-2.3511s}. \end{array}$$

**Figure 1:**
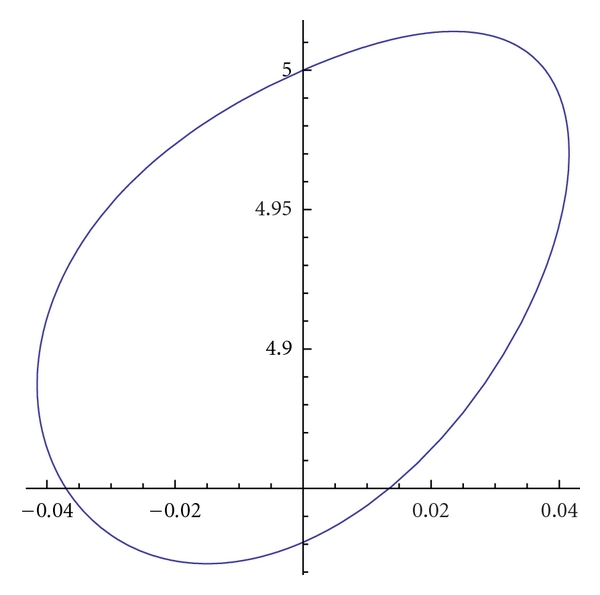


Using boundary conditions, we obtain the graph of the curve (*γ*) in Figure 1.

**Example 2**

If we choose in (12) *w*_1_ = 1, *w*_2_ = 1, *k*(*v*) = 10*v*, $I\left(v\right)=I\left(x,y\right)=(2\sqrt{2}/3)\left(x^{3/2}+y^{3/2}\right)$, *λ* = 1, and the boundary conditions *v*(0) = (1,1)^*T*^, *v*′(0) = (3.1,−8.1)^*T*^, *v*(*π*) = (1−*π*/10,−2−*π*/10−3cosh⁡⁡(2*π*))^*T*^, *v*′(*π*) = (2.1 − cosh⁡⁡(2*π*), −8.1 − 2sinh⁡⁡(2*π*)), then the (ELP) equations have the form:

(16) $$\begin{array}{c} x^{\text{iv}}-x^{\prime \prime }-10y^{\prime }-1=0,\\ y^{\text{iv}}-y^{\prime \prime }+10x^{\prime }-1=0, \end{array}$$

with the analytical solutions:

(17) $$\begin{array}{cc} x\left(s\right) & =\sin2s+\cos2s+\sin s\cosh2s+0.1s,\\ y\left(s\right) & =-4\sin2s+4\cos2s+3\cos s\cosh2s,\\ & \;-4\sinh2s\sin s-0.1s-6. \end{array}$$

**Figure 2:**
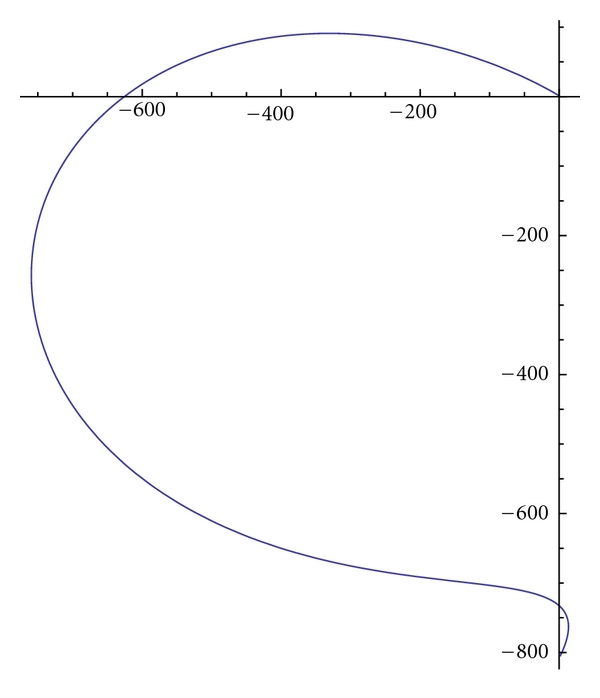


The graph of the curve (*γ*) is given in Figure 2.

### 2.2. Deformable Dynamic 2D Models

Roughly speaking, the differential fourth-order vectorial equation (11) or the differential eight-order system (12) may have many solutions, which leads to many possible energy-minimizing snakes. As we have seen in the preceding examples (Section 2.1), these solutions have a complicated form; moreover, they are often inaccessible explicitly. In order to eliminate these drawbacks, we point out in this section, two approaches which lead to a practical and more simple solution of the (ELP) equation.

#### 2.2.1. The Method of Evolution Equation [19]

Denote by

(18) $$\left(\gamma \right):v=v_{0}\left(s\right),\;0\leq s\leq 1,$$

an initial estimate of the optimal snake and let consider a family of curves (contours)

(19) $$\left(\gamma^{t}\right):v=v\left(t,s\right),\;v\in C^{2}\left({\mathbb{R}}_{+}\times \left[0,1\right],{\mathbb{R}}^{2}\right),$$

where the parameter *t* ≥ 0 describes the evolution in time of the snake and *s* ∈ [0,1] is the standard parameter of the curve. The *evolution equation* associated to the dynamic model is

(20) $$\begin{array}{cc} & \frac{\partial v}{\partial t}+\frac{\partial^{2}}{\partial s^{2}}\left(w_{2}\left(s\right)\frac{\partial^{2}v}{\partial s^{2}}\right)-\frac{\partial }{\partial s}\left(w_{1}\left(s\right)\frac{\partial v}{\partial s}\right)\\ & \;\;-J_{2}\left(\nabla k\right)\frac{\partial v}{\partial s}+\nabla P\left(v\right)-\left(\nabla k\right)\left(J_{2}v^{\prime }\right)=0, \end{array}$$

together with the initial condition:

(21) $$v\left(0,s\right)=v_{0}\left(s\right),\;0\leq s\leq 1,$$

and the boundary conditions

(22) $$\begin{array}{c} v\left(t,0\right)=v_{0}\left(0\right),\;v\left(t,1\right)=v_{0}\left(1\right),\\ \frac{\partial v}{\partial s}\left(t,0\right)=v_{0}^{\prime }\left(0\right),\;\frac{\partial v}{\partial s}\left(t,1\right)=v_{0}^{\prime }\left(1\right), \end{array}\;t\geq 0.$$

A solution of the *static* problem described by (ELP) equation (11) is achieved when the solution *v*(*t*, *s*) becomes stable with respect to the time parameter, that is, lim⁡_*t*→*∞*_(∂*v*/∂*t*)(*t*, *s*) = 0, uniformly with respect to the parameter *s* ∈ [0,1]; in this case, the evolution equation (20) provides a solution of the *static* problem (11). According to [20], we note that this approach of making the time derivative term vanish is equivalent to applying a gradient descent algorithm to find the local minimum of the energy functional *E*(*v*).

#### 2.2.2. The Method of the Lagrange Dynamics [3, 18]

A dynamic snake is represented by introducing a time-varying contour

(23) $$v\left(t,s\right)={\left(x\left(t,s\right),y\left(t,s\right)\right)}^{T},$$

see (19), with a mass density *μ*(*s*) and a damping density *ω*(*s*). The Lagrange equation for a snake defined in Section 2.1 is

(24) $$\begin{array}{cc} & \mu \frac{\partial^{2}v}{\partial t^{2}}+\omega \frac{\partial v}{\partial t}+\frac{\partial^{2}}{\partial s^{2}}\left(w_{2}\left(s\right)\frac{\partial^{2}v}{\partial s^{2}}\right)-\frac{\partial }{\partial s}\left(w_{1}\left(s\right)\frac{\partial v}{\partial s}\right)\\ & \;\;+\nabla P\left(v\right)-J_{2}\left(\nabla k\right)\frac{\partial v}{\partial s}-\left(\nabla k\right)\left(J_{2}v^{\prime }\right)=0. \end{array}$$

The first two terms in the left hand side of (24) represent the inertial and damping forces, while the remaining terms, see also (11), represent the internal stretching force (the term containing ∂*v*/∂*s*), the bending (rigidity) force (the term containing ∂^2^ *v*/∂*s* ^2^), the external force (∇*P*(*v*)) and the *balloon*-type force (the last two terms). Equilibrium is achieved when these forces balance and the contour comes to rest, that is,

(25) $$\frac{\partial v}{\partial t}=\frac{\partial^{2}v}{\partial t^{2}}=0,$$

which leads to the equilibrium condition (11).

### 2.3. Deformable Surfaces (3D Models)

In this section we define briefly the notion of deformable 3D model (deformable surface), both in the static and dynamic forms, and we describe a method for reducing the problem of its optimization to a 2D modelling problem.

#### 2.3.1. Energy-Minimizing Surfaces

From mathematical point of view, a 3D variational deformable model is emphasized by a family *𝒜* of parameterized smooth surfaces with given boundary conditions, named *admissible surfaces*, and an associated *energy functional*.

Denoting by *D* = [0,1] × [0,1] the unit square of ℝ^2^, let us consider a surface of vectorial equation:

(26) (S):v=v(s,r), (s,r)∈D,

where *v* ∈ *C* ^2^(*D*, ℝ^3^), *v* = (*x*,*y*,*z*)^*T*^; in this subsection we set |*v*|^2^ = *x* ^2^ + *y* ^2^ + *z* ^2^, *v*_*s*_ = ∂*v*/∂*s*, *v*_*ss*_ = ∂^2^ *v*/∂*s* ^2^, *v*_*sr*_ = ∂^2^ *v*/∂*s*∂*r*, *v*_*rr*_ = ∂^2^ *v*/∂*r* ^2^. Given the functions *g* ∈ *C* ^2^(∂*D*, ℝ^3^) and *h* ∈ *C* ^1^(∂*D*, ℝ^3^), where ∂*D* is the boundary of *D*, let *𝒜* be the set of *admissible deformations*, which consists of all functions *v* ∈ *C* ^2^(*D*, ℝ^3^) satisfying the boundary conditions *v*(*s*, *r*) = *g*(*s*, *r*) and (∂*v*/∂*n*)(*s*, *r*) = *h*(*s*, *r*) on ∂*D*, where *n* is the normal vector with respect to the surface (*S*) defined by (26). Further, let us consider the following functions: *the image intensity function I* ∈ *C* ^2^(ℝ^3^); *the potential function associated* to the external forces *P*(*v*) = −*λ*|∇*I*(*v*)|^2^, *λ* > 0; *the control functions* corresponding to the internal forces acting on the shape of the surface, namely, the elasticity functions *w*_10_(*s*; *r*) and *w*_01_(*s*; *r*); the *rigidity functions w*_20_(*s*; *r*) and *w*_02_(*s*; *r*), and *the twist resistance function w*_11_(*s*; *r*). The energy functional *E* : *𝒜* → ℝ, associated to these data, is defined as follows:

(27) $$E\left(v\right)=\iint_{D}^{}F\left(v,v_{s},v_{r},v_{ss},v_{sr},v_{rr}\right)\text{ds}\;\text{dr},$$

where

(28) $$\begin{array}{cc} F\left(v,v_{s},v_{r},v_{ss},v_{sr},v_{rr}\right) & =w_{10}{\left|v_{s}\right|}^{2}+w_{01}{\left|v_{r}\right|}^{2}\\ & \;+w_{20}{\left|v_{ss}\right|}^{2}+2w_{11}{\left|v_{sr}\right|}^{2}\\ & \;+w_{02}{\left|v_{rr}\right|}^{2}+f\left(v,v_{s},v_{r}\right),\\ f\left(v,v_{s},v_{r}\right) & =P\left(v\right)+\det\left(c_{0}v,v_{s},v_{r}\right). \end{array}$$

We notice that *E*(*v*) represents the sum of *the internal energy* (the terms of (27) excepting *f*(*v*, *v*_*s*_, *v*_*r*_)), *the external energy* (defined by the term containing *P*(*v*)) and *the balloon energy*, which is added, optionally, by the users (the term including det⁡(*c*_0_ *v*, *v*_*s*_, *v*_*r*_)).

The triple (*𝒜*, *I*, *E*) is said to be a *3D deformable model*, sometimes a *deformable surface*. The basic problem of the deformable model is to minimize its energy functional, namely, to obtain the optimal deformable surface. To this purpose, the Euler-Gauss-Ostrogradski (EGO) equation of Calculus of Variations, that is,

(29) $$\begin{array}{c} \frac{\partial F}{\partial v}-\frac{\partial }{\partial s}\left(\frac{\partial F}{\partial v_{s}}\right)-\frac{\partial }{\partial r}\left(\frac{\partial F}{\partial v_{r}}\right)+\frac{\partial^{2}}{\partial s^{2}}\left(\frac{\partial F}{\partial v_{ss}}\right)\\ \;\;+\frac{\partial^{2}}{\partial v\partial r}\left(\frac{\partial F}{\partial v_{sr}}\right)+\frac{\partial^{2}}{\partial r^{2}}\left(\frac{\partial F}{\partial v_{rr}}\right)=0 \end{array}$$

is used.

By simple calculation, we obtain from (28) and (29):

(30) $$\begin{array}{cc} & \frac{\partial^{2}}{\partial s^{2}}\left(w_{20}v_{ss}\right)+\frac{\partial^{2}}{\partial r^{2}}\left(w_{02}v_{rr}\right)+2\frac{\partial^{2}}{\partial s\partial r}\left(w_{11}v_{sr}\right)\\ & \;\;-\frac{\partial }{\partial s}\left(w_{10}v_{s}\right)-\frac{\partial }{\partial r}\left(w_{01}v_{r}\right)\\ & \;\;+\frac{1}{2}\left(\nabla f-\frac{\partial }{\partial s}\left(\frac{\partial f}{\partial v_{s}}\right)-\frac{\partial }{\partial r}\left(\frac{\partial f}{\partial v_{r}}\right)\right)=0. \end{array}$$

#### 2.3.2. Deformable Dynamic 3D Models

Similarly to the 2D model, we can suppose that a rough prior estimate of surface is accessible, namely,

(31) $$\left(S^{0}\right):v=v_{0}\left(s,r\right),\;\left(s,r\right)\in D.$$

Further, this surface is refined step by step, according to (EGO) equation; so, a sequence of surfaces, which leads to the energy-minimizing surface, is provided. More exactly, let

(32) $$\left(S^{t}\right):v=v\left(t,s,r\right),\;t\geq 0,\;\left(s,r\right)\in D,$$

be a family of surfaces, where the parameter *t* describes the evolution in time of the model. We associate to the previous static model (*𝒜*, *I*, *E*) the *evolution equation*

(33) $$\frac{\partial v}{\partial t}+G\left(v,v_{s},v_{r},v_{ss},v_{sr},v_{rr}\right)=0,$$

where *G*(*v*, *v*_*s*_, *v*_*r*_, *v*_*ss*_, *v*_*sr*_, *v*_*rr*_) is the left hand member of (30), together with the* initial estimate (condition)*

(34) $$v\left(0,s,r\right)=v_{0}\left(s,r\right),\;\left(s,r\right)\in D,$$

and the boundary dynamic conditions

(35) $$\begin{array}{c} v\left(t,s,r\right)=v_{0}\left(s,r\right),\;\left(s,r\right)\in \partial D,\;t\geq 0,\\ \frac{\partial v\left(t,s,r\right)}{\partial n}=\frac{\partial v_{0}\left(s,r\right)}{\partial n},\;\left(s,r\right)\in \partial D,\;t\geq 0. \end{array}$$

A solution of the “static” problem described by (30) is achieved, when the solution *v*(*t*, *s*, *r*) becomes stable with respect to the time parameter, that is, lim⁡_*t*→*∞*_(∂*v*/∂*t*)(*t*, *s*, *r*) = 0, uniformly, with respect to (*s*, *r*) ∈ *D*; in this case, the evolution equation (33) provides a solution of the static problem (30).

#### 2.3.3. The Simplified 2D Model

The problem of finding directly energy-minimizing surfaces, that is, solutions of the p.d.e. (30), is not practically possible because these solutions contain long and complicated expressions or their explicit form is inaccessible. On the other hand, by using discretized schemes for solving (33), we get a system of algebraic equations with a high computational level. These drawbacks are eliminated by passing to a 2D modeling problem, [19]. More exactly, the third component *z* of (*S*) is constrained to depend only on *r*, by setting *z*(*s*, *r*) = *r*. So, the surface that we seek is given as a sequence of plane curves, named *slices*, and the parameter *r* of (26) becomes the index of the corresponding slice. In this approach, the surface that we seek is viewed as a sequence of the planar curves (slices), indexed by the parameter *r*, so that each fixed value of *r* provides a closed curve, lying in a slice of the 3D-image. Consequently, let

(36) $$\left(\gamma_{r}\right):v\left(s\right)=\left(x\left(s\right),y\left(s\right)\right),\;s\in \left[0,1\right],$$

be the 2D curve obtained by applying this reconstruction method, for a given *r*.

Under the hypothesis that *w*_*ij*_ are positive constants, the (EGO) equation (29), which corresponds to (*γ*_*r*_), is

(37) $$2w_{20}\frac{d^{4}v}{ds^{4}}-2w_{10}\frac{d^{2}v}{ds^{2}}-c_{0}J_{2}\frac{dv}{ds}+\nabla P=0,$$

where $J_{2}=\left[\begin{array}{cc} 0 & 1\\ -1 & 0 \end{array}\right]$.

**Figure 3:**
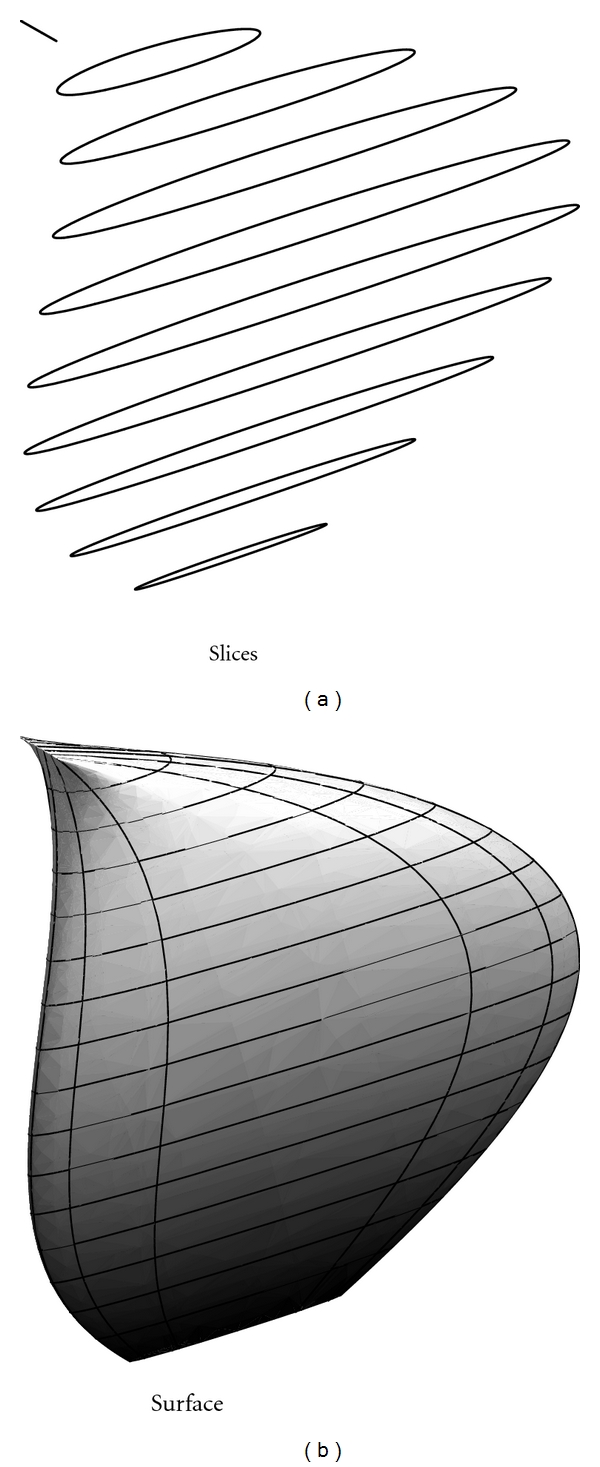


If we consider in (37) *c*_0_ = −0.02, *w*_10_ = 2.5, *w*_20_ = 0.4, *P* = *r*(*x* ^2^ + *y* ^2^) and *r* = 0.1,0.2,…, 1 with boundary conditions *x*(0) = *x*(1) = 1 + (*r* ^2^(1 − *r*)^2^)/25, *x*′(0) = *x*′(1) = *r*(1 − *r*)/20, *y*(0) = *y*(1) = 0 + *r* ^2^(1 − *r*)/25, *y*′(0) = *y*′(1) = 2*r*(1 − *r*)/5, we obtain the graphs of the slices and a 3D reconstruction of the surface, as we can see in Figures 3(a) and 3(b).

In what follows we shall restrict to the study of 2D deformable models.

## 3. An ELP-Algorithm for Obtaining Energy-Minimizing Snakes

In this section we suppose that the following hypotheses are satisfied: the control functions *w*_1_ and *w*_2_ are positive constants, the curves of the family (*γ* ^*t*^) given by (18) and (19) are closed for every *t* ≥ 0 and *k*(*v*) = *c*_0_ *v*, *c*_0_ ∈ ℝ_+_. Thus, the (ELP) evolution equation (20) becomes

(38) $$2\frac{\partial v}{\partial t}+2w_{2}\frac{\partial^{4}v}{\partial s^{4}}-2w_{1}\frac{\partial^{2}v}{\partial s^{2}}-2c_{0}J_{2}\frac{\partial v}{\partial s}+\nabla P=0,\;v=v\left(t,s\right).$$

In order to solve numerically the partial differential equation (38), we focus on the method of finite differences, which is widely used in image processing [21]. Let *δ* and *h* be the time and the space discretization steps, respectively, and denote by *ℛ* = {(*t*_*k*_, *s*_*i*_), *k* ≥ 0,0 ≤ *i* ≤ *N*} the plane net of discretization, with *N* ∈ ℕ*, *Nh* = 1, *t*_*k*_ = *kδ*, and *s*_*i*_ = *ih*. The following notations will be used, too: *v*_*i*_^*k*^ = *v*(*t*_*k*_, *s*_*i*_), *v* ^*k*^ = (*x* ^*k*^, *y* ^*k*^)^*T*^, *k* ≥ 0; *g* ^*k*^ = (*g*_1_^*k*^,*g*_2_^*k*^)^*T*^, with *g*_1_^*k*^ = (−1/2)((∂*P*/∂*x*)(*v* ^*k*^)), *g*_2_^*k*^ = (−1/2)((∂*P*/∂*y*)(*v* ^*k*^)); obviously, *v*_*i*_^*k*^ = *v*_*i*+*N*_^*k*^, *i* ∈ *ℤ*, because (*γ* ^*t*^),*t* ≥ 0, is a closed curve. Also, we set

(39) $$\alpha =\frac{w_{1}}{h^{2}},\;\;\beta =\frac{w_{2}}{h^{4}},\;\;\gamma =\frac{c_{0}}{h}.$$

### 3.1. Explicit Finite Difference Scheme

We approximate the partial derivatives involved in the (ELP) evolution equation (38) as follows:

(40) $$\begin{array}{c} \frac{\partial v}{\partial t}\left(t_{k},s_{i}\right)\approx \frac{1}{\delta }\left(v_{i}^{k+1}-v_{i}^{k}\right);\;\;\frac{\partial v}{\partial s}\left(t_{k},s_{i}\right)\approx \frac{1}{h}\left(v_{i+1}^{k}-v_{i}^{k}\right),\\ \frac{\partial^{2}v}{\partial s^{2}}\left(t_{k},s_{i}\right)\approx \frac{1}{h^{2}}\left(v_{i+1}^{k}-2v_{i}^{k}+v_{i-1}^{k}\right),\\ \frac{\partial^{4}v}{\partial s^{4}}\left(t_{k},s_{i}\right)\approx \frac{1}{h^{4}}\left(v_{i+2}^{k}-4v_{i+1}^{k}+6v_{i}^{k}-4v_{i-1}^{k}+v_{i-2}^{k}\right). \end{array}$$

By replacing the relations (40) in the partial differential equation (38), it result a system of algebraic equations; denoting by *V* ^*k*^ = (*X* ^*k*^,*Y* ^*k*^)^*T*^ the solutions of this system (which approximate the exact values *v*_*i*_^*k*^ of (38) at the nodes of *ℛ*), we get the vectorial formula:

(41) $$\begin{array}{cc} & \frac{V_{i}^{k+1}-V_{i}^{k}}{\delta }+\beta \left(V_{i+2}^{k}-4V_{i+1}^{k}+6V_{i}^{k}-4V_{i-1}^{k}+V_{i-2}^{k}\right)\\ & \;\;-\alpha \left(V_{i+1}^{k}-2V_{i}^{k}+V_{i-1}^{k}\right)\\ & \;\;-\gamma J_{2}\left(V_{i+1}^{k}-V_{i}^{k}\right)+\frac{1}{2}\nabla P=0,\;0\leq i\leq N;\;k\geq 0, \end{array}$$

where

(42) $$V_{i}^{k}=\left(X_{i}^{k},Y_{i}^{k}\right)$$

and *α*, *β*, *γ* are given by (39). The scalar equations corresponding to (41) are the following:

(43) $$\begin{array}{cc} & \frac{X_{i}^{k+1}-X_{i}^{k}}{\delta }+\beta \left(X_{i+2}^{k}-4X_{i+1}^{k}+6X_{i}^{k}-4X_{i-1}^{k}+X_{i-2}^{k}\right)\\ & \;\;-\alpha \left(X_{i+1}^{k}-2X_{i}^{k}+X_{i-1}^{k}\right)\\ & \;\;-\gamma \left(Y_{i+1}^{k}-Y_{i}^{k}\right)\\ & \;\;+\frac{1}{2}\frac{\partial P}{\partial x}\left(X_{i}^{k},Y_{i}^{k}\right)=0,\;0\leq i\leq N-1;\;k\geq 0.\\ & \frac{Y_{i}^{k+1}-Y_{i}^{k}}{\delta }+\beta \left(Y_{i+2}^{k}-4Y_{i+1}^{k}+6Y_{i}^{k}-4Y_{i-1}^{k}+Y_{i-2}^{k}\right)\\ & \;\;-\alpha \left(Y_{i+1}^{k}-2Y_{i}^{k}+Y_{i-1}^{k}\right)\\ & \;\;+\gamma \left(X_{i+1}^{k}-X_{i}^{k}\right)\\ & \;\;+\frac{1}{2}\frac{\partial P}{\partial y}\left(X_{i}^{k},Y_{i}^{k}\right)=0,\;0\leq i\leq N-1;\;k\geq 0. \end{array}$$

Now, let *K* be the *stiffness matrix* associated to the explicit finite difference scheme, defined as the circular matrix of order *N*, whose first row is

(44) $$\left(a_{1},a_{2},a_{3},0,\ldots ,0,a_{3},a_{2}\right),$$

where

(45) $$a_{1}=2\alpha +6\beta ,\;\;a_{2}=-\alpha -4\beta ,\;\;a_{3}=\beta .$$

Denote by *L* the circular (square) matrix of order *N* defined by the first row (1, −1,0, 0,…, 0) and let *I*_*N*_ be the identity matrix of order *N*. The relations (41) and (43) can be written in a matricial form as:

(46) $$\begin{array}{c} V^{k+1}=\left(I_{N}-\delta K\right)V^{k}-\gamma \delta L\left(J_{2}V^{k}\right)+\delta g^{k},\;k\geq 0, \end{array}$$

 

(47) $$\begin{array}{c} X^{k+1}=\left(I_{N}-\delta K\right)X^{k}-\gamma \delta LY^{k}+\delta g_{1}^{k},\\ Y^{k+1}=\left(I_{N}-\delta K\right)Y^{k}+\gamma \delta LX^{k}+\delta g_{2}^{k}, \end{array}\;k\geq 0$$

respectively.

In what follows, the formulas (41)–(47) will be referred as *(ELP) algorithm* for obtaining an energy minimizing snake (in its approximating form).

### 3.2. The Residue of (ELP) algorithm

Taking into account the relation (41), the residue associated to the (ELP) algorithm is

(48) $$\begin{array}{cc} Rv_{i} & =\frac{v_{i}^{k+1}-v_{i}^{k}}{\delta }+\beta \left(v_{i+2}^{k}-4v_{i+1}^{k}+6v_{i}^{k}-4v_{i-1}^{k}+v_{i-2}^{k}\right)\\ & \;-\alpha \left(v_{i+1}^{k}-2v_{i}^{k}+v_{i-1}^{k}\right)-\gamma J_{2}\left(v_{i+1}^{k}-v_{i}^{k}\right)\\ & \;+\frac{1}{2}\nabla P\left(v_{i}^{k}\right),\;0\leq i\leq N,\;k\geq 0. \end{array}$$

By using Taylor expansions at the point (*t*_*k*_, *s*_*i*_) ∈ *ℛ* we obtain

(49) $$\begin{array}{cc} v_{i}^{k+1} & =v_{i}^{k}+\delta \frac{\partial v}{\partial t}+\frac{\delta^{2}}{2!}\frac{\partial^{2}v}{\partial t^{2}}+\frac{\delta^{3}}{3!}\frac{\partial^{3}v}{\partial t^{3}}+\cdots ,\\ v_{i\pm 1}^{k} & =v_{i}^{k}\pm h\frac{\partial v}{\partial s}+\frac{h^{2}}{2!}\frac{\partial^{2}v}{\partial s^{2}}\pm \frac{h^{3}}{3!}\frac{\partial^{3}v}{\partial s^{3}}+\cdots ,\\ v_{i\pm 2}^{k} & =v_{i}^{k}\pm 2h\frac{\partial v}{\partial s}+\frac{{\left(2h\right)}^{2}}{2!}\frac{\partial^{2}v}{\partial s^{2}}\pm \frac{{\left(2h\right)}^{3}}{3!}\frac{\partial^{3}v}{\partial s^{3}}+\cdots , \end{array}$$

where the partial derivatives ∂*v*/∂*t* and ∂^*l*^ *v*/∂*s* ^*l*^, *l* ≥ 1 are computed at the point (*t*_*k*_, *s*_*i*_) = (*kδ*, *ih*) ∈ *ℛ*.

By replacing the expansions (49) in the residue's formula (48) and using the relations (39), we derive

(50) $$\begin{array}{cc} Rv_{i} & =\delta \left(\frac{1}{2}\frac{\partial^{2}v}{\partial t^{2}}+\frac{\delta }{6}\frac{\partial^{3}v}{\partial t^{3}}+\cdots \right)\left(t_{k},s_{i}\right)\\ & \;+h^{2}w_{2}\left(\frac{1}{6}\frac{\partial^{6}v}{\partial s^{6}}+\frac{127}{5040}h^{2}\frac{\partial^{8}v}{\partial s^{8}}+\cdots \right)\left(t_{k},s_{i}\right)\\ & \;-w_{1}h^{2}\left(\frac{1}{12}\frac{\partial^{4}v}{\partial s^{4}}+\frac{h^{2}}{60}\frac{\partial^{6}v}{\partial s^{6}}+\cdots \right)\left(t_{k},s_{i}\right)\\ & \;-c_{0}J_{2}h\left(\frac{1}{2}\frac{\partial^{2}v}{\partial v^{2}}+\frac{h^{2}}{24}\frac{\partial^{4}v}{\partial v^{4}}+\cdots \right)\left(t_{k},s_{i}\right),\;\\ & \;\;\;\;\;\;\;\;\;\;0\leq i\leq N-1,\;k\geq 0. \end{array}$$

If the partial derivatives of the vectorial function *v* are uniformly bounded on *D*, the relations (50) give the following estimate concerning the residue of (ELP) algorithm:

(51) $$Rv_{i}=\{\begin{array}{cc} O\left(\delta \right)+O\left(h\right), & \text{if}\;c_{0}>0,\\ O\left(\delta \right)+O\left(h^{2}\right), & \text{if}\;c_{0}=0. \end{array}$$

Notice that the condition *c*_0_ = 0 means that there are not existing constrains defined by the users.

### 3.3. The Consistency of the ELP algorithm

Let *Tr*⁡(*v*_*i*_) = *δRv*_*i*_ be *the truncature error *of (ELP) algorithm at the *k*th iteration. Under the assumption of uniform boundedness of the partial derivatives of the vectorial function *v*, it follows from (51):

(52) $$\mathrm{Tr}\left(v_{i}\right)=\{\begin{array}{cc} O\left(\delta^{2}\right)+O\left(\delta h\right), & \text{if}\;c_{0}>0\\ O\left(\delta^{2}\right)+O\left(\delta h^{2}\right), & \text{if}\;c_{0}=0. \end{array}$$

The relations (52) characterize the accuracy of the discretized scheme providing the (ELP)-Algorithm.

On the other hand, the equality

(53) $$\underset{\begin{array}{c} \delta \to 0\\ h\to 0 \end{array}}{\lim}\frac{\mathrm{Tr}\left(v_{i}\right)}{\delta }=\underset{\begin{array}{c} \delta \to 0\\ h\to 0 \end{array}}{\lim}\;Rv_{i}=0,$$

which results from (52), shows that this discretized scheme is consistent.

### 3.4. Approximation Error and the Convergence

Let us consider the approximation-error *ε*_*i*_^*k*^ at the point (*t*_*k*_, *s*_*i*_) ∈ *ℛ*, namely

(54) $$\varepsilon_{i}^{k}=v_{i}^{k}-V_{i}^{k},\;0\leq i\leq N-1,\;k\geq 0.$$

By replacing *V*_*i*_^*k*^ = *v*_*i*_^*k*^ − *ε*_*i*_^*k*^ from (54) into (41) and taking into account the expressions (49) and the definition (48) of *Rv*_*i*_, we get

(55) $$\begin{array}{cc} \varepsilon_{i}^{k+1} & =\delta Rv_{i}-\beta \delta \varepsilon_{i+2}^{k}+\delta \left(\left(4\alpha +\beta \right)I_{2}+\gamma J_{2}\right)\varepsilon_{i+1}^{k}\\ & \;+\left(\left(1-6\beta \delta -2\alpha \delta \right)I_{2}-\gamma J_{2}\right)\varepsilon_{i}^{k}\\ & \;+\delta \left(4\beta +\alpha \right)\varepsilon_{i-1}^{k}-\beta \delta \varepsilon_{i-2}. \end{array}$$

Let

(56) $$E^{k}=\max\left\{\left|\varepsilon_{i-2}^{k}\right|,\left|\varepsilon_{i-1}^{k}\right|,\left|\varepsilon_{i}^{k}\right|,\left|\varepsilon_{i+1}^{k}\right|,\left|\varepsilon_{i+2}^{k}\right|\right\},\;k\geq 0,$$

be the *approximation error* of (ELP) algorithm at *k*th iteration.

The relations (55) and (56) yield:

(57) $$\begin{array}{cc} E^{k+1} & \leq \delta \left|Rv_{i}\right|\\ & \;+(\beta \delta +\delta \sqrt{{\left(4\beta +\alpha \right)}^{2}+\gamma^{2}}\\ & \;\;\;\;+\sqrt{{\left(1-6\beta \delta -2\alpha \delta \right)}^{2}+\gamma^{2}\delta^{2}}+4\beta \delta +\alpha \delta +\beta \delta )E^{k}. \end{array}$$

On the other hand, it follows from (50):

(58) $$\left|Rv_{i}\right|\leq M_{1}\delta +\left|2w_{2}-w_{1}\right|M_{2}h^{2}+c_{0}M_{3}h,\;0\leq i\leq N-1,$$

where *M*_*j*_, *j* ≥ 1 are positive constants, which do not depend on *δ* and *h*.

Now, the relations (57) and (58), combined with the classic inequality

(59) $$\sqrt{x^{2}+y^{2}}\leq \left|x\right|+\left|y\right|,$$

provide the estimate:

(60) $$\begin{array}{cc} E^{k+1} & \leq \left(10\beta \delta +2\alpha \delta +2\gamma \delta +\left|1-6\beta \delta -2\alpha \delta \right|\right)E^{k}+A\left(h,\delta \right), \end{array}$$

with

(61) $$A\left(h,\delta \right)=M_{1}\delta^{2}+M_{2}\left|2w_{2}-w_{1}\right|\delta h^{2}+M_{3}c_{0}\delta h.$$

Denote by

(62) $$\varepsilon =\frac{\delta }{h^{4}}$$

and let us assume that the inequality

(63) $$6\varepsilon w_{2}\left(k+1\right)\leq 1$$

holds. It is a simple exercise to show that the relation (63) entails the inequality

(64) 6βδ+2αδ≤1

for *N* sufficiently large. Now, the relations (60) and (64) lead to:

(65) $$E^{k+1}\leq qE^{k}+A\left(h,\delta \right),\;k\geq 0,$$

where

(66) $$E^{0}=0,\;\;q=1+4\beta \delta +2\gamma \delta .$$

Writing (65) successively for *k*, *k* − 1,…, 1, we get

(67) $$E^{k+1}\leq \frac{q^{k+1}-1}{q-1}A\left(h,\delta \right),\;k\geq 0.$$

Taking into account that *γ* ≤ *β* (for *N* sufficiently large), the relations (66) and (39) imply 1 + 4*w*_2_ *ε* ≤ *q* ≤ 1 + 6*w*_2_ *ε*, so that the relations (67) and (63), combined with the inequality (1+*x*)^1/*x*^ ≤ *e*, *x* > 0, yield

(68) $$E^{k+1}\leq \frac{e-1}{4w_{2}\varepsilon }A\left(h,\delta \right).$$

Finally, we derive from (61), (62), and (68):

(69) $$E^{k+1}=\{\begin{array}{cc} O\left(\delta h^{4}\right)+O\left(h^{5}\right), & \text{if}\;c_{0}>0,\\ O\left(\delta h^{4}\right)+O\left(h^{6}\right), & \text{if}\;c_{0}=0. \end{array}$$

It follows from (69) that *E* ^*k*+1^ → 0 if *h* → 0; it is easily seen that, according to the relations (62) and (63), the hypothesis *h* → 0 implies *δ* → 0; consequently the following result holds.

*If the inequality (63) fulfills, then the (ELP) algorithm (46) is convergent and its approximation error at the *(*k* + 1)*th iteration is given by the relation (69).*

### 3.5. The Stability

The intuitive idea regarding the stability is that small errors in the initial conditions of a partial differential equation should cause small errors in its solution. In fact, the study of the stability is useful in connection with the theorem of Lax concerning the convergence of the discretized schemes, [21].

The aim of this subsection is to examine the stability of the (ELP) algorithm (46), with *c*_0_ = 0. By omitting the small terms *δRv*_*i*_ of (55), we get the relation:

(70) $$\begin{array}{cc} \varepsilon_{i}^{k+1} & =\left(1-6\beta \delta -2\alpha \delta \right)\varepsilon_{i}^{k}+\left(\alpha \delta +4\beta \delta \right)\left(\varepsilon_{i+1}^{k}+\varepsilon_{i-1}^{k}\right)\\ & \;-\beta \delta \left(\varepsilon_{i+2}^{k}+\varepsilon_{i-2}^{k}\right),\;k\geq 0. \end{array}$$

To apply the *stability criterion of von Neumann*, [22] we set

(71) $$\begin{array}{c} r_{1}=\alpha \delta ,\;\;r_{2}=\beta \delta ,\\ \eta_{1}=\omega_{1}h,\;\;\eta_{2}=\omega_{2}h, \end{array}\;\;$$

(72) $$\begin{array}{cc} \varepsilon_{i}^{k} & =\exp\left(\nu kl\right)\exp\left(j\omega h\right)\\ & ={\left(\mu^{k}e^{j\omega_{1}ih},\mu^{k}e^{j\omega_{2}ih}\right)}^{T},\\ \mu & =\exp\left(\nu l\right), \end{array}$$

where *j* and *ν* = *ν*(*ω*) are complex numbers, *j* ^2^ = −1 and *ω* = (*ω*_1_,*ω*_2_)^*T*^ denotes the frequency.

Now, we obtain from (70), (71), and (72):

(73) $$\begin{array}{cc} \mu & =\left(1-2r_{1}-6r_{2}\right)+\left(r_{1}+4r_{2}\right)\left(e^{j\eta_{1}}+e^{-j\eta_{1}}\right)\\ & \;-r_{2}\left(e^{2j\eta_{1}}+e^{-2j\eta_{1}}\right),\\ \mu & =\left(1-2r_{1}-6r_{2}\right)+\left(r_{1}+4r_{2}\right)\left(e^{j\eta_{2}}+e^{-j\eta_{2}}\right)\\ & \;-r_{2}\left(e^{2j\eta_{2}}+e^{-2j\eta_{2}}\right). \end{array}$$

We choose *ω*_1_ = *ω*_2_ and let *η* = *η*_1_/2 = *η*_2_/2. The trigonometric formulas *e* ^*jα*^ + *e* ^−*jα*^ = 2cos⁡⁡*α*, 1 − cos⁡⁡*α* = 2sin⁡^2^ *α*/2, and 1 − cos⁡⁡2*α* = 8 sin⁡^2^ *α*/2 cos⁡^2^ *α*/2, *α* ∈ ℝ together with (73) give:

(74) $$\mu =1-4r_{1}\sin^{2}\eta -16r_{2}\sin^{4}\eta .$$

On the other hand, according to the relation |exp⁡(*jωit*)| = 1, it is easy to see that the error *ε*_*i*_^*k*^ of (72) does not increase in time if |*μ*| ≤ 1, so that we infer from (74):

(75) $$2r_{1}+8r_{2}\leq 1,$$

which represent precisely the *stability criterion of von Neumann* for the (ELP) algorithm (46).

A combination of the relations (39), (62), (71), and (75) provides the following equivalent form of the stability condition of von Neumann:

(76) $$2\varepsilon \left(4w_{2}+w_{1}h^{2}\right)\leq 1.$$

## 4. Monitoring the Behavior of Prosthetic Surgical Methods and Prosthetic Medical Materials Based on Software Implementation

In order to apply the results of the theoretical researches detailed above in the medical imaging domain, a 3D visual software environment—named MoDef—was implemented, aiming to visualize and follow up the deformation behavior of the surgical (abdominal, maxilla-facial, and orthodontic) prosthetic materials. That is performed on three distinct, but convergent, levels, as follows:

- 3D reconstruction visual software component, aimed to tracks the evolution of the prosthetic materials, based on processing the US images of the anatomic context of a lot of surgical patients;
- deformable prosthetic material's behavior forecasting software component, based on software tools which implements the above described mathematical methods;
- quad comparative parallel tracking software component, aimed to simultaneous supervise in time both (a) and (b) levels, in comparison with the results provided by the stochastic analysis component of the 3D visual software environment MoDef.

Concerning the 3D visualizing of the prosthetic meshes by means of the MoDef software environment components, two levels of reconstruction are performed, namely

1. on the first level, a polynomial interpolation method is applied on each slice of the US image of the prosthetic mesh, acquired based on succeeding positions of the transducer, obtained by rotating them with a constant angle in a same preestablished direction; more exactly, the curves representing the sections of the surgical mesh acquired by the transducer are extracted from the context of the US image, based on specific image processing methods, namely, contour detection methods, that are implemented at the level of the image processing operators of the MoDef environment's image processing library. Starting with this set of basic mesh surface definition curves, extracted from the US images acquired at pre-established moments in time, a complete and consistent collection of 3D generator curve sets is obtained, by means of 3D polynomial interpolation methods, based on Lagrange, Hermite or Birkhoff operators;
2. on the second level, the complete collection of the 3D generator curves obtained at the first level is processed based on Blended Interpolating Methods (BIM), as well as with 3D continuous representation techniques, in order to obtain “solid-view,” respectively, “wired-view” representations of the prosthetic mesh.

**Figure 4:**
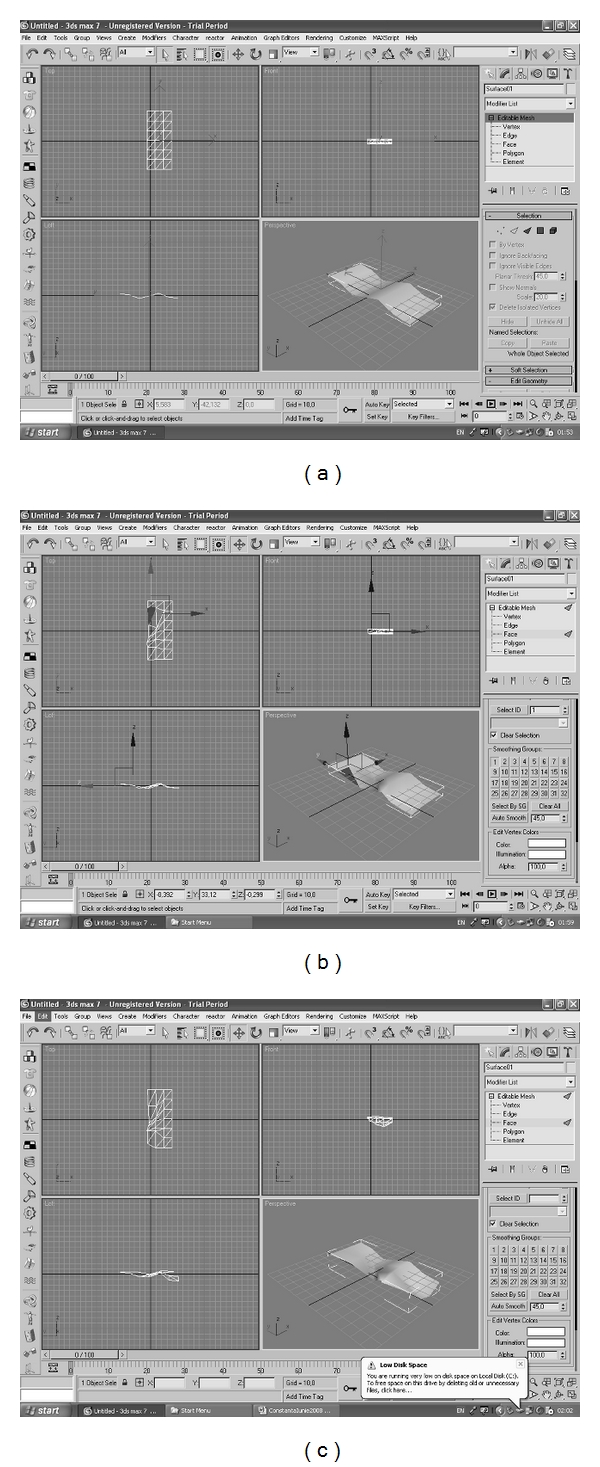
Preliminary experiments made in 3DS Max7.

**Figure 5:**
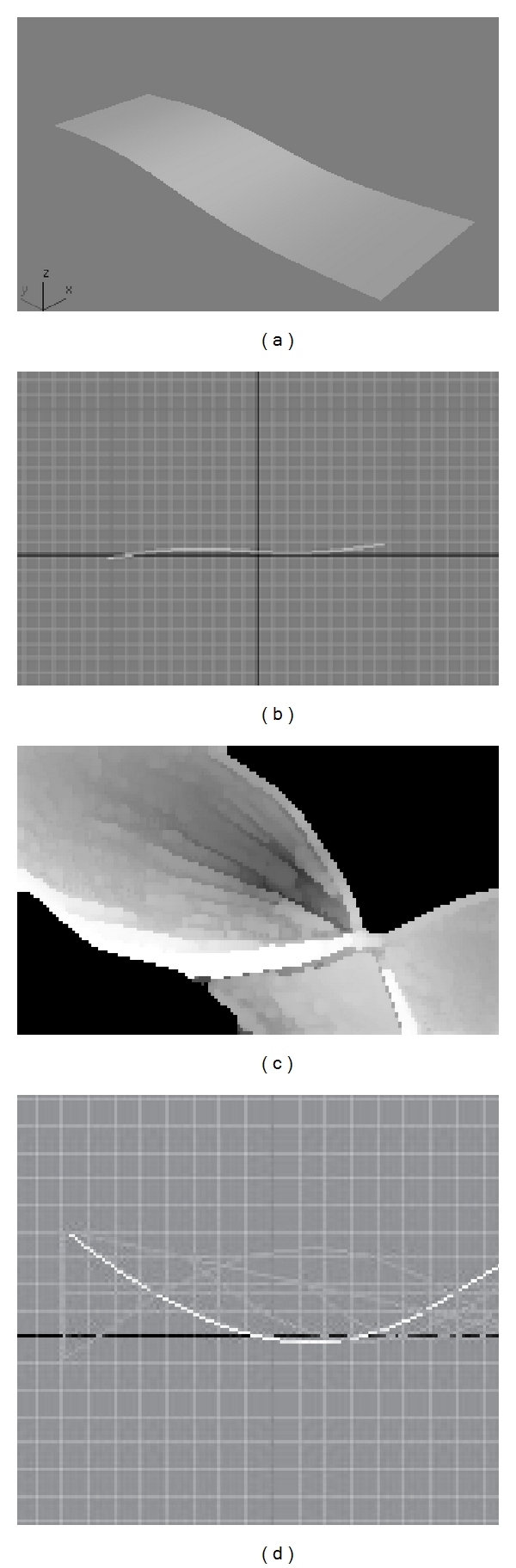
Results obtained with the 3D reconstruction component of MoDef 3D Visual environment: (a) initial 3D representation of the deformable surface of the surgical mesh, (b) curve representing a section of the surgical mesh acquired by the transducer, extracted from the context of the US image based on specific-image processing method, (c) the surface of the prosthetic mesh after the deformations produced in time due to the anatomic assimilation process, and (d) the basic set of generating curves, used to obtain the solid-view representations of the prosthetic mesh.

In what follows, some preliminary experiments made in 3DS Max7, followed by some relevant results obtained with the 3D reconstruction component of MoDef 3D Visual environment are presented in Figures 4 and 5.

## 5. Conclusions

In this paper we considered parametric (variational) deformable models and we developed an iterative method based on finite difference schemes in order to solve numerically the (ELP) equation of Calculus of Variations, which provides the energy minimizing snake. We derived estimates concerning the approximation error related to the corresponding (ELP) algorithm and we established conditions for its convergence and stability. Some considerations about the implementation of the above numerical methods where presented, too. As future targets, we intend to consider probabilistic models which offer an alternative approach by using the Bayes technique, as well as geometric deformable models which provide an efficient alternative to address some limitation of parametric deformable models.
